# High risk alcohol-related trauma among the Aboriginal and Torres Strait Islanders in the Northern Territory

**DOI:** 10.1186/1747-597X-7-33

**Published:** 2012-08-03

**Authors:** Rama Jayaraj, Mahiban Thomas, Valerie Thomson, Carolyn Griffin, Luke Mayo, Megan Whitty, Peter d’Abbs, Tricia Nagel

**Affiliations:** 1 Wellbeing and Preventable Chronic Diseases Division, Menzies School of Health Research and School of Environmental and Life Sciences, Charles Darwin University, Darwin, Northern Territory, Australia; 2 Department of Head and Neck Surgery, Royal Darwin Hospital, Darwin, Northern Territory, Australia; 3 Wellbeing and Preventable Chronic Diseases Division, Menzies School of Health Research, Darwin, Northern Territory, Australia

**Keywords:** Facial trauma, Indigenous Australians or Aborginal and Torres Strait Islanders, Alcohol related injury, Culturally appropriate intervention

## Abstract

High risk drinking is linked with high rates of physical harm. The reported incidence of alcohol - related trauma among Aboriginal and Torres Strait Islander people in the Northern Territory is the highest in the world. Facial fractures are common among young Aboriginal and Torres Strait Islanders. They are often linked with misuse of alcohol in the Northern Territory and are frequently secondary to assault. This review focuses on alcohol-related trauma in the Territory and draws attention to an urgent need for preventative health approach to address this critical issue.

## Introduction

While the Northern Territory (NT) of Australia is acclaimed for its unique flora and fauna and pristine outback wilderness, it faces public health concerns that need urgent attention. One of these is the high risk drinking of its population. Per capita alcohol consumption and alcohol attributable death rates in the NT are markedly higher than Australia generally, while alcohol attributable hospitalisations are more than twice the national rate [1]. The most common alcohol-related problems encountered in the NT include trauma, assault, delinquent behaviour, interpersonal violence and facial injury [2]. Facial trauma frequently require surgical treatment [3] and occurs at an extremely high rate and is most frequently the result of interpersonal violence linked with alcohol in the NT [4]. Furthermore, the incidence of mandibular fracture is the highest in the Territory, and is second only to Greenland worldwide [4]. Aboriginal and Torres Strait Islander people, who comprise 32% (66,600) of the NT population [5] , are at much greater risk of engaging in high risk drinking and are more vulnerable to alcohol -related injury and death [6,7]. This paper explores the incidence of alcohol - related harm in the NT population and proposes treatment strategies including brief intervention and culturally adopted intervention in a health framework.

## Alcohol - related harm

Alcohol has been linked to one third of all self harm injuries and suicides in Australia [8]. Young people in particular are at high risk of injuries [7] and the percentage of alcohol-related deaths among Aboriginal and Torres Strait Islander youth aged 15–24 years is estimated to be almost three times higher than for non-Indigenous youth [9]. Additionally, alcohol and other drugs are major contributing factors to the high percentage of road injuries of NT Aboriginal and Torres Strait Islander people [10] and injuries secondary to assault are of particular concern.

### Assault

Victims of assault that were using alcohol at the time of the initial injury are at much greater risk of recurrent trauma in non-Indigenous population [11]. High risk alcohol consumption is also linked with family conflict, domestic violence and assaults among Aboriginal and Torres Strait Islander people [12,13]. Aboriginal and Torres Strait Islander people have higher rates of injury, hospitalisation and death caused by assault than other Australians [14,15] and in the NT non-metropolitan areas levels of alcohol-caused assaults and hospitalisations are much higher than elsewhere in Australia [2]. There were a total of 6678 assaults recorded during 2009 to 2010 and sixty percent were alcohol-related [16]. Furthermore, 52% of family violence incidents and 36% of reported incidents requiring police attendance were alcohol-related, highlighting the strong link between alcohol and violent crime [17]. Additionally, it has been shown that the majority of assaults against women in remote NT communities are perpetrated by a drunken husband or other family member. Such alcohol related crime is one of the factors leading to the vast overrepresentation of Aboriginal and Torres Strait Islander people in the NT prisons (82% of the daily average prison population) [18].

### Alcohol - related facial trauma

Facial injury is a particularly common outcome of alcohol-related violence in the NT. Sixty percent of all facial fractures seen in the NT occur in Aboriginal and Torres Strait Islander patients and more than 90% are the result of assault [4]. The incidence of mandibular fractures in Aboriginal and Torres Strait Islander people in the NT is reported to be 155 per 100,000 of population, while facial fractures in the NT overall were close to 120 per 100,000 of population [4]. Thomas and Scott (2009) report that of 236 consecutive patients with facial fracture, 88% of injuries are linked with alcohol intoxication and all were secondary to assault. Facial fractures have also been shown to increase seasonally in the NT linked with increasing day time humidity and temperature [3]. Alarmingly, recurrence rates of facial trauma in the NT are the highest in the world and 96% of recurrent facial trauma occurs in Aboriginal and Torres Strait Islander people [3].

### Estimated costs of alcohol associated harm, crime and trauma in the NT

The cost of alcohol’s harms to others in the Australian society is estimated at approximately $20 billion each year [19]. The cost per person of alcohol-related harms in the NT is more than four times the national level [20]. Collins and Lapsley calculated the costs of alcohol, tobacco and illicit drug abuse to Australian society in 2004/05 as equivalent to $934 per person. The comparative cost in the NT was $4,197 [21]. It is not only physical injury and disability which results from high risk drinking, but associated emotional distress and increased risk of mental illness such as post traumatic stress disorder [22,23].

## Preventive measures

There is clearly a need to explore preventive strategies to address this major public health issue. The recommendations within current health preventative strategies [24] aim to shift the culture of alcohol through regulation, legislation, health promotion and treatment services. In the NT there has been recent focus on legislative changes through new alcohol reforms [25] which seek to reduce the per capita consumption of alcohol (currently the highest in the country) to the national average. The new and amended legislation to support the ‘Enough is Enough’ Alcohol Reform includes introduction of a ‘Banned Drinker Register’, Alcohol Court Reform and the establishment of an Alcohol and Other Drugs Tribunal, training and resource provision for health care providers across the Territory, and communication and awareness campaigns [25].

In addition, the Northern Territory Alcohol Policing Strategy, which complements the Northern Territory’s 2030 Strategy, [26] supports a range of activities such as night patrolling services across the Territory [27]. These patrols are designed to maintain community security and safety through transfer of intoxicated people to sobering-up shelters or rehabilitation services [27]. The NT Alcohol framework [28] and NT Liquor Act under review at present [29] are important other avenues with potential to prevent harm. Legislation is only one aspect of a suite of strategies needed. One of the priority areas of the *Expanding Health Service Delivery Initiative (EHSDI),* a joint Australian and Northern Territory Government initiative seeking to close the gap in Aboriginal and Torres Strait Islander disadvantage, is to enhance access to alcohol interventions through primary and acute health care services [30].

### Treatment

There are several approaches to the treatment of alcohol use disorders. Approaches include family therapy, cognitive-behavioural interventions, motivational interviewing, pharmacological treatments, and Alcoholics Anonymous [31]. Many of these are conducted in specialised centres such as rehabilitation clinics, which are separated from the main public hospital centres. Hospitalisation for alcohol-related trauma and injury provides an opportunity to enhance access through identification and referral of individuals with underlying alcohol problems requiring treatment. Admission to a hospital ward is preferable: this is presumably, “as an opportunity for brief intervention” [32]. Hospitilisation for alcohol-related trauma in the maxillofacial or trauma ward also creates an opening for motivating patients to review their alcohol consumption at a time when their facial injury may make them more receptive to advice. However as patients with alcohol-related injuries are often treated in maxillofacial surgery unit, where mental health is not a primary focus and the surgical workload is often great, such opportunities for intervention are frequently overlooked.

Clinical staff and health professionals need to be appropriately trained to explore appropriate intervention/treatment strategy and referral pathways for trauma patients with alcohol misuse in the hospital setting. A common misconception by clinical staff in the health care setting is to believe that only a specialist can handle patients with alcohol misuse, such is the nature of a highly compartmentalised public hospital system. There is also a need for increasing research in this area. Researchers need to train in intervention research methodology and also the funding bodies that are being urged to fund alcohol intervention studies in order to increase the research workforce in this field [33]. Legislative amendment and media involvement are also pivotal in enhancing the work of treatment services based at the hospital setting to reduce the alcohol-related harms that are currently faced by society [34].

### Brief interventions

Brief interventions have been shown to be effective with mild symptoms of alcohol dependency and non-dependent drinkers for reducing alcohol intake, risky drinking practices, alcohol-related negative consequences and injury frequency [35-38]. Miller and Sacnhez describe 6 common elements to a brief intervention, summarized by the acronym FRAMES; FEEDBACK of personal risk or impairment, emphasis on personal RESPONSIBILITY for change, clear ADVICE to change, a MENU of alternative change options, therapeutic EMPATHY as a counseling style, and enhancement of client SELE-EFPICACY or optimism [39]. Mutiple randomized trials have shown a significant reduction in alcohol consumption with the use of brief interventions in appropriately targeted non-Indigenous populations in a variety of health care settings, provided the patients are non- dependent drinkers [36,40,41].

The compelling evidence of association between alcohol misuse, injury and reinjury has led the American College of Surgeons to recommend routine screening and brief behavioural interventions for all trauma admissions [42]. The computerized tailored brief intervention is associated with a significant decrease in alcohol use and at-risk drinking in sub-critically injured trauma patients [43]. However, while there is some evidence of effectiveness of these strategies in hospital settings [44], it remains inadequate [45]. The training and support initiatives continue to show no significant effects on uptake by staff, prompting calls for systematic approaches to implementing brief intervention in the hospital setting [45]. Four key elements underlie health-care practitioner’s perceptions of alcohol screening and brief intervention; outcome expectancy; role congruence; utilisation of clinical systems and processes; and options for alcohol referral [46]. Despite this work, there is a dearth of studies that examine intervention strategies for Indigenous Australians: there is atleast one study [47], albeit unsuccessful.

There is general agreement that brief interventions have positive outcomes for patients and generally comply with the time constraints and the cost conscious environment of the hospital. However, in the context of Australian Indigenous patients, brief interventions may not be culturally appropriate and effective in their traditional format.

## Culturally adapted interventions

Adapting strategies to the cultural needs of Abroginal and Torres Strait Islander people is especially important given differences of worldview, literacy and language [48]. Direct questions, for example, are a common component of screening tools and may be perceived as culturally inappropriate [47,49]. International research suggests that racial and ethnic disparities in alcoholism treatment exist in terms of access to, appropriateness, and quality of care and that these differences have not been adequately explored [47]. In the United States a small study tested the effectiveness of a culturally adapted brief intervention (Motivational Enhancement Therapy) for use with 25 Native Americans [50]. The study found that those receiving Motivational Enhancement Therapy had better drinking outcomes than those in the Twelve Step Facilitation approach (an approach that helps people to link with Alcoholics Anonymous) [50]. Although this was only a small study the authors concluded that the adapted intervention seemed to be a helpful approach [51].

In Australia, there have been no studies exploring culturally adapted interventions for high risk drinking in the hospital setting. There has however been a recent study in the NT primary care setting. A brief structured psychological intervention, ‘Motivational Care Planning’ was tested, using a randomised controlled design, in the setting of comorbid alcohol dependence and mental illness and found to be effective [52-54]. The intervention (two 1 - hour sessions) was developed with Aboriginal Mental Health Workers in three remote communities through the Australian Integrated Mental health initiative (AIMhi) [55]. Motivational Care Planning (MCP) shares theoretical and practical facets with a range of brief therapies such as motivational interviewing, problem solving therapy, and solution focused therapy [56,57].

Motivational Care Planning focuses on family and culture and uses metaphors such as trees and football playing. These metaphors resonate strongly with Aboriginal and Torres Strait Islander people. The research team translated their findings into culturally adapted tools and training addressing comorbid mental health and alcohol misuse [57]. These culturally adapted resources are now in use in a number of primary care, mental health, chronic disease, and alcohol misuse services across Australia [58-61]. There is a high need for further robust studies which test the effectiveness of early intervention strategies for Aboriginal and Torres Strait Islander people.

## Conclusion

The costs of alcohol- related injuries to the Australian community are high: whether experienced directly by the victim through physical injury and emotional suffering, or indirectly through the distress caused to families and the loss of productivity in the community. The impact of high risk drinking is particularly evident in the NT. Further investigation of measures to diminish the ongoing impact of alcohol misuse on the health and wellbeing of trauma victims and their families is essential. Such measures must include a comprehensive range of actions: policy committed to harm minimisation, accompanying health promotion and awareness campaigns, and a suite of treatment options from opportunistic brief interventions in acute care settings to structured treatment delivered by a spectrum of service providers from primary care to specialist in Alcohol and other Drug services.

## Abbreviations

NT: Northern Territory; AIMhi: Australian Integrated Mental health initiative; MCP: Motivational Care Planning.

## Competing interests

The authors declared that they have no competing interest.

## Authors’ contributions

JR and MT conceived of the study, participated in its design and coordination, carried out the review, and drafted the manuscript. VT, CG, LM and Pd conceived of the study, participated in its design and helped draft the manuscript. MW performed the literature search and helped draft the manuscript. TN conceived of the study, participated in its design and helped draft the manuscript. All authors read and approved the final manuscript.
